# Genomic Regions Associated with Resistance to Gastrointestinal Nematode Parasites in Sheep—A Review

**DOI:** 10.3390/genes15020187

**Published:** 2024-01-30

**Authors:** Samla Marques Freire Cunha, Stephanie Lam, Bonnie Mallard, Niel A. Karrow, Ángela Cánovas

**Affiliations:** 1 Centre for Genetic Improvement of Livestock, Department of Animal Biosciences, University of Guelph, 50 Stone Rd E, Guelph, ON N1G 2W1, Canada; scunha@uoguelph.ca (S.M.F.C.); slam02@uoguelph.ca (S.L.); bmallard@ovc.uoguelph.ca (B.M.); nkarrow@uoguelph.ca (N.A.K.); 2 Department of Pathobiology, University of Guelph, 50 Stone Rd E, Guelph, ON N1G 2W1, Canada

**Keywords:** candidate genes, genomics, genome-wide association study, gastrointestinal nematodes, sheep, quantitative trait loci

## Abstract

Gastrointestinal nematodes (GINs) can be a major constraint and global challenge to the sheep industry. These nematodes infect the small intestine and abomasum of grazing sheep, causing symptoms such as weight loss, diarrhea, hypoproteinemia, and anemia, which can lead to death. The use of anthelmintics to treat infected animals has led to GIN resistance, and excessive use of these drugs has resulted in residue traced in food and the environment. Resistance to GINs can be measured using multiple traits, including fecal egg count (FEC), Faffa Malan Chart scores, hematocrit, packed cell volume, eosinophilia, immunoglobulin (Ig), and dagginess scores. Genetic variation among animals exists, and understanding these differences can help identify genomic regions associated with resistance to GINs in sheep. Genes playing important roles in the immune system were identified in several studies in this review, such as the *CFI* and *MUC15* genes. Results from several studies showed overlapping quantitative trait loci (QTLs) associated with multiple traits measuring resistance to GINs, mainly FEC. The discovery of genomic regions, positional candidate genes, and QTLs associated with resistance to GINs can help increase and accelerate genetic gains in sheep breeding programs and reveal the genetic basis and biological mechanisms underlying this trait.

## 1. Introduction

Gastrointestinal nematodes (GINs), also known as ‘roundworms’, are a major constraint and challenge to the global sheep industry [1,2]. The most clinically and economically relevant species infecting sheep include *Haemonchus contortus*, *Teladorsagia circumcincta*, *Trichostrongylus* spp. (predominantly *Trichostrongylus colubriformis*), *Cooperia curticei*, and *Oesophagostomum* spp. [3]. Nematodes infect the small intestine and abomasum of grazing sheep, and animals can develop detrimental symptoms such as weight loss, diarrhea, hypoproteinemia, and anemia [3,4,5] and can die of infection if left untreated [6]. GIN infection is an animal welfare issue, and producers can face substantial economic losses that are difficult to measure. Expected losses are attributed to treatments, management strategies, and veterinary care. These losses are minor compared to losses of reduced production (milk and wool), weight loss, and mortality, also attributed to GIN infection [7]. One of the major approaches to preventing GIN infection is the use of anthelmintics to treat infected animals. However, overreliance on anthelmintics has led to the development of GIN resistance to the available drugs [8,9,10] and anthelminthic residues in food and the environment [9,10,11].

Resistance to GINs can be measured using a wide range of traits [9,12,13]. The most common trait used to measure resistance to GINs in sheep is fecal egg count (FEC) [8,12,14]. This trait is moderately heritable, and when worms are present, animals with a small number of eggs per gram are considered desirable [9,15,16,17]. Other traits can be used to measure resistance in sheep, such as FAffa MAlan CHArt (FAMACHA©) scores (scores from 1—paler to 5—pink), hematocrit (HCT), also known as packed cell volume (PCV), eosinophilia, immunoglobulin (Ig) levels, and accumulation of feces in the wool in the breech area, known as dagginess scores (dag score; 1—no dags to 5—extensive dags from the breech area to the hocks) [12,13,18,19]. Traits such as HCT and PCV are highly laborious to measure as they entail blood collection and laboratory analysis when compared to FAMACHA© scores, which require the measurement of the ocular conjunctival mucous membrane color. In addition, these traits characterize levels of anemia that can also be a physiological response not related to GIN infection [5]. However, they can indicate infection caused by hematophagous parasites, like *H. contortus*, and have shown value in determining optimal management strategies and serving as selection traits, particularly for flocks where parasite burden is prevalent [9,19]. Other traits used to measure GIN resistance, especially for scientific purposes, are total and antigen-specific immunoglobulin (Ig) levels (e.g., IgA, IgE, and IgG), abomasum and small intestine worm count assessed at necropsy, and plasma protein levels [9].

Researchers have confirmed differences in the immune response among GIN-infected animals, which can be attributed to genetic variations among animals [20,21,22]. By discovering and understanding the underlying genetic regions contributing to the variation in individual immune response to GIN infection and resistance to GIN infection in sheep, breeding strategies can be tailored to mitigate the impact of these parasites [22].

One approach to understanding the genetic basis of traits is performing a genome-wide association study (GWAS) to identify significant genetic markers, more recently single nucleotide polymorphisms (SNPs) associated with a trait. Recent studies have conducted a GWAS for resistance to GINs in sheep using SNP panels [6,23,24,25,26,27,28,29,30,31,32]. However, in the early 2000s, before the availability of SNP panels, studies performing association analysis used microsatellite markers [33,34,35,36,37,38,39]. Several results have been found regarding genomic regions, candidate genes, and quantitative trait loci (QTLs) associated with several traits related to resistance to GINs in sheep. In addition to the number of results produced in past years, little consensus has been achieved regarding the major genes and similar genomic regions affecting traits related to resistance to GINs in different sheep breeds or populations [1,6,23].

The range of results reported in the literature may be explained by several reasons: (i) resistance to gastrointestinal nematodes is a complex and polygenic trait with no major or singular gene contributing to the phenotype [40,41]; (ii) the type of methodology and statistical models used to identify regions associated with the trait vary among studies [1]; (iii) the type of markers used in the association analysis (50K SNP array—Ovine SNP50 array or 50K SNP BeadChip array, high-density array, customized SNP panel, whole genomic sequence, or microsatellites); (iv) the type of nematode infecting the animals (*T. circumcincta*, *Trichostrongylus* spp., *H. contortus*., or mixed species) [1,22]; (v) the type of infection (naturally exposed or artificially challenged); (vi) the different breeds used in the study, including resistant or susceptible genetically selected lines, and different cross experiments (backcross or F1) [1]; (vii) the different traits used to measure resistance (e.g., FAMACHA©, PCV, FEC, IgA or IgE levels, among others); (viii) the phase of the infection when the animals were evaluated; (ix) the age, sex, and physiological stage (pregnant, lactating, lamb) of the evaluated animals; (x) the size of the population and the number of animals with genotypic and phenotypic records; and (xi) the geographical location of the animal population.

The discovery of genomic regions, positional candidate genes, QTLs, and markers associated with resistance to GINs can help increase and accelerate genetic gains in breeding programs and reveal the genetic basis and biological mechanisms involved with sheep resistance to GINs [24]. However, the above-described reasons resulting in the variability of results reported in the literature highlight major challenges to interpreting the genetic basis of resistance to GINs and in formulating breeding strategies to enhance resistance and must be taken into consideration moving forward. This review summarizes the results and studies in the literature that aimed to identify genes or QTLs associated with resistance to GINs in sheep.

## 2. Association Studies for Resistance to Gastrointestinal Nematodes in Sheep

**Figure 1:**
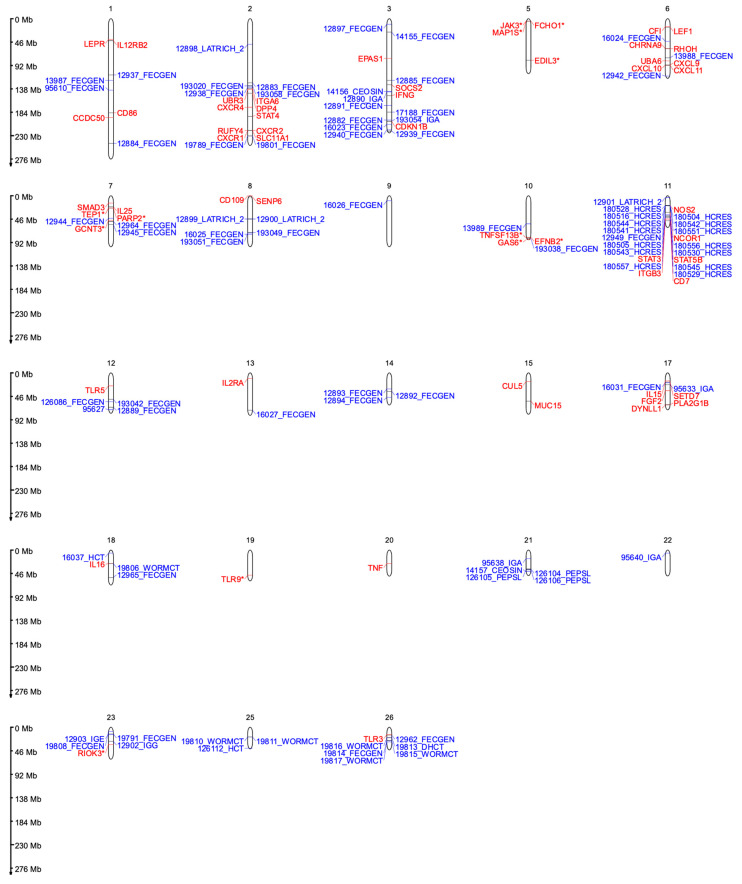
Genes (in red) and quantitative trait loci (QTLs) (in blue) identified as overlapping among genome-wide association studies and association analyses for each ovine chromosome. The genes and QTLs were associated with different traits measuring resistance to gastrointestinal nematodes. The positions were retrieved from the NCBI and the Sheep Animal QTL Database using the Oar_v3.1 sheep assembly. * Genes were described in the main text based on the positions presented in their reference study, which diverges from the recently updated version of the sheep assembly used to generate this figure.

Genomic regions associated with resistance to GIN infection in sheep were found across all chromosomes, and several genes and QTLs have been identified in these regions by several authors. This review focused on genes and QTLs that overlapped between two or more studies. Considering that a substantial number of regions overlapped within the same study (different methods and/or different traits) and between studies, as well as the large number of results identified by all studies, only the genes and QTLs related to the immune system response to GIN infection or resistance to GINs in sheep were further described. The QTLs were identified by their ID number as described in the Sheep Animal QTL Database [42] “https://www.animalgenome.org/cgi-bin/QTLdb/OA/index (accessed on 5 December 2023)”. All genes and QTLs described in this study are summarized in Table 1 and Table 2, respectively. Gene and QTL locations on their respective chromosomes, based on the Oar_v3.1 sheep assembly, can be visualized in Figure 1 (MG2C version 2.1 [43]).

### 2.1. Chromosome 1

Several papers spanned different genomic regions, candidate genes, and QTLs on OAR 1 for different traits related to resistance to GIN infection in sheep. The *coiled-coil domain containing 50* (*CCDC50*) gene was identified by Benavides et al. [6] in a GWAS for FEC average in a double backcross population of Red Maasai and Dorper sheep breeds and by Berton et al. [30] in a single-step GWAS (ssGWAS) analysis associated with FAMACHA©, HCT, and red blood cell count (RBC) traits using haplotype information in a Santa Inês sheep population. The *CCDC50* gene in humans may function as a negative regulator of NF-κB signaling that plays an important role in the regulation of inflammatory responses [48,49,50].

Estrada-Reyes et al. [44] performed a signature of selection study and identified the *cd86 molecule* (*CD86*) gene in a region under selection in the comparison between Katahdin and St. Croix breeds. Additionally, this gene was identified in a GWAS associated with RBC, platelets (PLT), and HCT in a naturally exposed Santa Inês sheep breed [29]. The *CD86* gene encodes a membrane protein that is expressed by antigen-presenting cells (APCs) and plays an important role in T-cell activation. When this protein binds to the cluster of differentiation 28 protein (CD28) on a T-cell during antigen presentation, it provides a co-stimulatory signal that helps activate T-cells to undergo proliferation and differentiation. During T-cell activation, membrane protein cytotoxic T-lymphocyte antigen-4 (CTLA-4; CD152) becomes induced, which has higher binding affinity for CD86, and its ligation with CD86 initiates an “off switch,” disengaging the T-cell from the APC [51].

Another important gene that may play a role in the defense against GIN infection is the *interleukin 12 receptor subunit β 2* (*IL12RB2*). This gene was identified by Berton et al. [29] as associated with PLT and HCT traits, by Estrada-Reyes et al. [41] as associated with FEC at 28 days and FAMACHA© traits, and by Estrada-Reyes et al. [45] as associated with IgM levels. Estrada-Reyes et al. [41] investigated SNPs inside a pre-selected panel of 100 genes related to the immune response to GINs that could be associated with eleven different traits measuring resistance/resilience to GINs in naturally infected Florida Native sheep with *H. contortus*. Similarly, Estrada-Reyes et al. [45] had the same objective of searching for SNPs associated with GIN resistance/resilience within 100 genes related to the immune response to GINs. However, the authors studied a different set of sheep breeds (Dorper, Katahdin, and St. Croix), and the animals were subjected to an artificial challenge and monitored for 42 days post-infection. The *IL12RB2* gene encodes a protein that is a subunit of the interleukin 12 receptor complex. Interleukin (IL) 12 and its receptor possess high affinity, and their binding is necessary to initiate signaling [52]. IL-12 is an important cytokine responsible for several processes within the immune response, and it is produced by dendritic cells in response to pathogen infection [53]. It plays a role in the regulation of T- and natural killer (NK) cells, differentiation of T helper 1 (Th1) cells, stimulation of interferon-γ (IFN-γ) production, and is related to antitumor and antiviral activity [53,54]. This gene was also found upregulated in human patients with the indeterminate form of chronic Chagas disease [55]. In addition, this gene is related to the immunological pathways “IL12 family signaling pathway,” “Th1 and Th2 cell differentiation route,” and “STAT1 proteins in the Jak-STAT signaling pathway network” [55].

Carracelas et al. [31] performed a ssGWAS analysis using a panel with 170 SNPs and identified a significant region associated with FEC in Corriedale sheep in which the *leptin receptor* (*LEPR*) gene is located. This gene was also identified by Berton et al. [29] as associated with RBC, PLT, and HCT phenotypes. The *LEPR* gene encodes a protein called the leptin hormone receptor that regulates body weight. Due to the similarity between the structure of leptin and the long-chain α-helical cytokine family and the receptors for interleukins and leptin, leptin can act as a cytokine, known as adipokine [56]. As an adipokine, leptin can regulate several functions of the innate immune system (e.g., increase IL-12 secretion in NK cells, regulate the activity and function of neutrophils, and induce the production of the pro-inflammatory cytokine IL-6) and adaptive immune system (e.g., promote proliferation of naïve T-cells and IL-2 secretion, increase the stimulation of Th1 cells, and stimulate proliferation, maturation, and survival of thymic T-cells) [56]. It is also important to notice that some infections may deregulate leptin levels and promote malnutrition, which may contribute to weight loss when animals are infected by GINs and other microbial infections [56].

Marshall et al. [57] performed a genome-wide scan to map QTLs using microsatellite markers associated with FEC after artificial challenges with *H. contortus* in Merino sheep. QTL 12937 was initially associated with FEC after the second artificial challenge [57] and was also associated with average FEC [19]. Pickering et al. [19] used SNP effects obtained by genomic prediction to find regions associated with the studied traits in a population composed of different breeds and crossbreeds naturally exposed to GINs. Considering this QTL was found to be associated with FEC in two different studies using different breeds and populations, this may be an important region for future studies.

QTL 13987 was identified by Gutiérrez-Gil et al. [58] as associated with FEC on day 60 after natural exposure to GINs in the Spanish Churra sheep breed. The authors mapped the QTL using the multimarket regression method for half-sib design within and across families. The same QTL was associated with the FEC of gastrointestinal nematodes using four different methods (logistic regression GWAS (LR-GWAS), population differentiation statistic (F_ST_), cross-population extended haplotype homozygosity (XP-EHH), and genome-wide distributions of runs of homozygosis (ROH)) in a study investigating genomic regions associated with variation in GIN infection (infected vs. non-infected animals) in naturally infected Tunisian sheep [24].

Atlija et al. [27], in a linkage disequilibrium and linkage analysis (LDLA) study in milking Spanish Churra ewes, revealed that QTL 95610 was associated with FEC. In addition, an SNP presenting a significant recessive effect with FEC at 28 days was identified by Estrada-Reyes et al. [59] within this QTL. Estrada-Reyes et al. [59] performed a GWAS focused on looking for additive and non-additive effects of genes associated with resistance to GINs in naturally exposed Florida Native sheep.

Lastly, QTL 12884 was revealed by Beh et al. [60] as associated with the average FEC of *T. colubriformis* after two rounds of artificial infection of Merino sheep. The authors performed a genome linkage scan using microsatellite information distributed around all chromosomes using interval analysis. The same QTL was identified inside a region associated with IgA levels [27].

**Table 2:** Quantitative trait loci (QTLs) associated with traits measuring resistance to gastrointestinal nematodes in sheep breeds identified as overlapping between association studies.

Chromosome	QTL ^1^	Associated Phenotype ^2^	Breed ^3^	Reference
1	12937	Average FEC	Merino	[57]
		EBV of the average FEC	Multiple breeds	[19]
	13987	FEC	Spanish Churra	[58]
		FEC	Tunisian	[24]
	95610	FEC	Spanish Churra	[27]
		FEC	Florida Native	[59]
	12884	*T. colubriformis* FEC	Merino	[60]
		Antigen-specific IgA activity	Spanish Churra	[27]
2	12898	*Trichostrongylus* spp. adults and late-stage larvae counts	Romney × Coopworth	[35]
		FEC	Tunisan	[24]
		FEC	Florida Native	[59]
		PCV	Djallonké	[25]
		FAMACHA©	Djallonké	[25]
		Resistance to GINs ^5^	Scottish Blackface	[46]
	193058	*Nematodirus* spp. average FEC	Scottish Blackface	[61]
		EBV of the average FEC	Multiple breeds	[19]
	193020	*Nematodirus* spp. FEC at 20 weeks of age	Scottish Blackface	[61]
		EBV of the average FEC	Multiple breeds	[19]
	12883	EBV of the average FEC	Multiple breeds	[19]
		*Nematodirus* spp. FEC at 20 weeks of age	Scottish Blackface	[36]
	12938	EBV of the average FEC	Multiple breeds	[19]
		Average FEC	Merino	[57]
	19801	FEC	Red Massai × Dorper	[38]
		Resistance to GINs ^5^	Scottish Blackface	[46]
	19789	FEC	Red Massai × Dorper	[38]
		Resistance to GINs ^5^	Scottish Blackface	[46]
3	12882	*Nematodirus* spp. FEC	Scottish Blackface	[36]
		EBV of the dag at 8 months	Multiple breeds	[19]
		FEC	Tunisian	[24]
	12885	*T. colubriformis* FEC	Merino	[60]
		Resistance to GINs ^5^	Scottish Blackface	[46]
	12890	Antigen-specific IgA activity	Scottish Blackface	[36]
		EBV of the FEC	Multiple breeds	[19]
		EBV of the FEC	Multiple breeds	[19]
	12891	Strongyle FEC	Scottish Blackface	[36]
		Antigen-specific IgA activity	Spanish Churra	[27]
	12897	FEC	Merino	[57]
		FEC	Tunisian	[24]
		Resistance to GINs ^5^	Scottish Blackface	[46]
	12939	FEC	Merino	[57]
		FEC	Florida Native	[59]
	12940	FEC	Merino	[57]
		FEC	Florida Native	[59]
		FEC	Florida Native	[59]
	14155	Average FEC	Merino × Romney	[37]
		FEC	Tunisian	[24]
	14156	Eosinophil level	Merino × Romney	[37]
		EBV of the FEC	Multiple breeds	[19]
		EBV of the FEC	Multiple breeds	[19]
	16023	Average FEC [45]	Red Massai × Dorper	[39]
		FEC	Tunisian	[24]
	17188	*Nematodirus* spp. FEC	Texel, Suffolk	[62]
		FEC	Tunisian	[24]
	193054	Antigen-specific IgA activity	Scottish Blackface	[61]
		EBV of the FEC	Multiple breeds	[19]
		EBV of the FEC	Multiple breeds	[19]
6	16024	Average FEC	Red Massai × Dorper	[39]
		FEC	Tunisian	[24]
		FEC	Spanish Churra	[27]
		Resistance to GINs ^5^	Scottish Blackface	[46]
	12942	FEC	Merino	[57]
		Average FEC	Multiple breeds	[19]
	13988	EBV of the FEC	Spanish Churra	[58]
		FEC	Spanish Churra	[27]
7	12944	FEC	Merino	[57]
		EBV of the FEC	Multiple breeds	[19]
	12945	FEC	Merino	[57]
		EBV of the FEC	Multiple breeds	[19]
	12964	FEC	Merino	[57]
		FEC	Spanish Churra	[27]
		EBV of the FEC	Multiple breeds	[19]
8	16025	Average FEC	Red Massai × Dorper	[39]
		Antigen-specific IgA activity	Spanish Churra	[27]
		FEC	Tunisan	[24]
	193049	Strongyles FEC	Scottish Blackface	[61]
		EBV of the dag at 8 months	Multiple breeds	[19]
	193051	Strongyles FEC	Scottish Blackface	[61]
		EBV of the dag at 8 months	Multiple breeds	[19]
	12899	Total counts of adults and late-stage larvae of *Trichostrongylus* spp. found in the abomasum	Romney × Coopworth	[35]
		FEC	Tunisan	[24]
		FEC	Djallonké	[25]
		PCV	Djallonké	[25]
		Antigen-specific IgA activity	Spanish Churra	[27]
		FAMACHA©	Florida Native	[59]
		Resistance to GINs ^5^	Scottish Blackface	[46]
	12900	Total counts of adults and late-stage larvae of *Trichostrongylus* spp. found in the small intestine	Romney × Coopworth	[35]
		FAMACHA©	Florida Native	[59]
		Antigen-specific IgA activity	Spanish Churra	[27]
		FEC	Tunisan	[24]
		Resistance to GINs ^5^	Scottish Blackface	[46]
		FEC	Djallonké	[25]
		PCV	Djallonké	[25]
9	16026	Average FEC	Red Massai × Dorper	[39]
		FEC	Spanish Churra	[27]
10	193038	Strongyles FEC	Scottish Blackface	[61]
		*H. contortus* FEC	Florida Native	[47]
		PVC	Florida Native	[47]
	13989	FEC	Spanish Churra	[58]
		FEC	Tunisan	[24]
		Antigen-specific IgA activity	Spanish Churra	[27]
		FEC	Florida Native	[59]
		Resistance to GINs ^5^	Scottish Blackface	[46]
11	180504	*H. contortus* resistance ^4^	Dorper, Katahdin, St. Croix	[44]
		FEC	Florida Native	[47]
	180505	*H. contortus* resistance ^4^	Dorper, Katahdin, St. Croix	[44]
		FEC	Florida Native	[47]
	180516	*H. contortus* resistance ^4^	Dorper, Katahdin, St. Croix	[44]
		FEC	Florida Native	[47]
	180528	*H. contortus* resistance ^4^	Dorper, Katahdin, St. Croix	[44]
		FEC	Florida Native	[47]
	180529	*H. contortus* resistance ^4^	Dorper, Katahdin, St. Croix	[44]
		FEC	Florida Native	[47]
	180530	*H. contortus* resistance ^4^	Dorper, Katahdin, St. Croix	[44]
		FEC	Florida Native	[47]
	180541	*H. contortus* resistance ^4^	Dorper, Katahdin, St. Croix	[44]
		FEC	Florida Native	[47]
	180542	*H. contortus* resistance ^4^	Dorper, Katahdin, St. Croix	[44]
		FEC	Florida Native	[47]
	180543	*H. contortus* resistance ^4^	Dorper, Katahdin, St. Croix	[44]
		FEC	Florida Native	[47]
	180544	*H. contortus* resistance ^4^	Dorper, Katahdin, St. Croix	[44]
		FEC	Florida Native	[47]
	180545	*H. contortus* resistance ^4^	Dorper, Katahdin, St. Croix	[44]
		FEC	Florida Native	[47]
	180551	*H. contortus* resistance ^4^	Dorper, Katahdin, St. Croix	[44]
		FEC	Florida Native	[47]
	180556	*H. contortus* resistance ^4^	Dorper, Katahdin, St. Croix	[44]
		FEC	Florida Native	[47]
	180557	*H. contortus* resistance ^4^	Dorper, Katahdin, St. Croix	[44]
		FEC	Florida Native	[47]
	12949	*H. contortus* FEC	Merino	[57]
		EBV of the FEC	Multiple breeds	[19]
		EBV of the FEC	Multiple breeds	[19]
	12901	Total counts of adults and late-stage larvae of *Trichostrongylus* spp. found in the small intestine	Romney × Coopworth	[35]
		FEC	Tunisian	[24]
		*H. contortus* FEC	Florida Native	[59]
		Resistance to GINs ^5^	Scottish Blackface	[46]
12	193042	*Nematodirus* spp. FEC	Scottish Blackface	[61]
		EBV of the FEC	Multiple breeds	[19]
	126086	EBV of the FEC	Multiple breeds	[19]
		*H. contortus* FEC	Martinik Black Belly × Romane	[63]
	95627	Antigen-specific IgA activity	Spanish Churra	[27]
		FEC	Tunisan	[24]
		Resistance to GINs ^5^	Scottish Blackface	[46]
	12889	*T. colubriformis* FEC	Merino	[60]
		Antigen-specific IgA activity	Spanish Churra	[27]
13	16027	Average FEC	Red Massai × Dorper	[39]
		FAMACHA©	Djallonké	[25]
14	12892	*Nematodirus* spp. FEC	Scottish Blackface	[36]
		FEC	Tunisan	[24]
		RBC	Santa Inês	[29]
	12893	*Nematodirus* spp. FEC	Scottish Blackface	[36]
		FEC	Tunisan	[24]
	12894	*Nematodirus* spp. FEC	Scottish Blackface	[36]
		Resistance to GINs ^5^	Scottish Blackface	[46]
15	16029	Average FEC	Red Masaai × Dorper	[39]
		Antigen-specific IgA activity	Spanish Churra	[27]
		EBV of the dag at 3 months	Multiple breeds	[19]
		EBV of the dag at 8 months	Multiple breeds	[19]
17	16031	Average FEC	Red Masaai × Dorper	[39]
		FEC	Spanish Churra	[27]
		Antigen-specific IgA activity	Spanish Churra	[27]
		Resistance to GINs ^5^	Scottish Blackface	[46]
	95633	Antigen-specific IgA activity	Spanish Churra	[27]
		FEC	Tunisan	[24]
		Resistance to GINs ^5^	Scottish Blackface	[46]
18	16037	PCV	Red Masaai × Dorper	[39]
		FAMACHA©	Djallonké	[25]
	19806	Number of adult worms found in the abomasum at necropsy	Red Masaai × Dorper	[38]
		FEC	Tunisan	[24]
	12965	*H. contortus* FEC	Merino	[57]
		Resistance to GINs ^5^	Scottish Blackface	[46]
21	14157	Eosinophil number	Merino × Romney	[37]
		FEC	Tunisan	[24]
		FEC	Spanish Churra	[27]
		Antigen-specific IgA activity	Spanish Churra	[27]
	95638	Antigen-specific IgA activity	Spanish Churra	[27]
		*H. contortus* FEC	Florida Native	[59]
	126104	Serum pepsinogen level	Martinik Black Belly × Romane,	[63]
		FEC	Tunisan	[24]
	126105	Serum pepsinogen level	Martinik Black Belly × Romane	[63]
		FEC	Tunisan	[24]
	126106	Serum pepsinogen level	Martinik Black Belly × Romane	[63]
		FEC	Tunisan	[24]
22	95640	Antigen-specific IgA activity	Spanish Churra	[27]
		FAMACHA©	Djallonké	[25]
23	19808	FEC	Red Masaai × Dorper	[38]
		Resistance to GINs ^5^	Scottish Blackface	[46]
		FEC	Santa Inês	[30]
		HCT	Santa Inês	[30]
		HGB	Santa Inês	[30]
		RBC	Santa Inês	[30]
	19791	FEC	Red Masaai × Dorper	[38]
		Resistance to GINs ^5^	Scottish Blackface	[46]
	12902	Total IgE level	Romney × Coopworth	[35]
		Antigen-specific IgA activity	Spanish Churra	[27]
	12903	Antigen-specific IgG level	Romney × Coopworth	[35]
		Antigen-specific IgA activity	Spanish Churra	[27]
		Resistance to GINs ^5^	Scottish Blackface	[46]
25	19810	Total counts of adult and immature worms at necropsy	Red Masaai × Dorper	[38]
		EBV of the FEC	Multiple breeds	[19]
	19811	Total count of adult worms at necropsy	Red Masaai × Dorper	[38]
		EBV of the FEC	Multiple breeds	[19]
	126112	PCV	Martinik Black Belly × Romane	[63]
		EBV of the FEC	Multiple breeds	[19]
26	19813	PCV	Merino	[57]
		EBV of the FEC	Multiple breeds	[19]
	19814	FEC	Merino	[57]
		EBV of the FEC	Multiple breeds	[19]
	19815	Total counts of adult and immature worms at necropsy	Merino	[57]
		EBV of the FEC	Multiple breeds	[19]
	19816	Total count of adult worms at necropsy	Merino	[57]
		EBV of the FEC	Multiple breeds	[19]
	19817	Total count of immature worms at necropsy	Merino	[57]
		EBV of the FEC	Multiple breeds	[19]
		EBV of the FEC	Multiple breeds	[19]
		EBV of the FEC	Multiple breeds	[19]
	12962	*H. contortus* FEC	Merino	[57]
		EBV of the FEC	Multiple breeds	[19]

^1^ The QTL number is the same as described in the Sheep Animal QTL Database “https://www.animalgenome.org/cgi-bin/QTLdb/OA/index, accessed on 5 December 2023)”. ^2^ The phenotypes with the same acronyms may differ between the experimental design, type of infection, nematode species, and other aspects; refer to the study or main text for more details. ^3^ “×” indicates a crossbreed study, and “,” indicates several breeds used in the study. ^4^ *H. contortus* resistance, as defined by reference [40], refers to a phenotype of fecal egg count after an artificial challenge with *H. contortus* used to identify regions under selection for resistance to gastrointestinal nematodes. ^5^ Resistance to GINs refers to the average of four replicates of fecal egg count that were collected at approximately 16, 20, and 24 weeks of age; however, the specific age at the time of sampling associated with the region was not specified by the authors. FEC—fecal egg count, IgA—immunoglobulin A, PCV—packed cell volume, FAMACHA©—FAffa MAlan CHArt©, GINs—gastrointestinal nematodes, dag: dagginness score, RBC—red blood cell, HCT—hematocrit, HGB—hemoglobin.

### 2.2. Chromosome 2

The OAR 2 was reviewed as a very prolific chromosome containing many genomic regions, genes, and QTLs that overlapped among several studies. Niciura et al. [40] performed a case-control GWAS for FEC and PCV collected at 21, 28, 35, and 42 days after artificial infection of *H. contortus* following two rounds of infection in Morada Nova sheep. The *integrin subunit α 6* (*ITGA6*) gene was found in a region associated with *H. contortus* resistance by Niciura et al. [40] and with PLT by Berton et al. [30]. This gene was also enriched (*p* < 0.05) in the gene ontology (GO) term “leukocyte migration” [30]. The levels of expression of the α6-integrin subunit protein produced by the *ITGA6* gene have an impact on T-cell adhesion to thymic epithelial cells and impact their migration within the thymus, development, and selection [64]. This integrin has also been implicated in the trafficking of circulating T-cells to the intestinal lamina propria [65].

The genes *c-x-c motif chemokine receptor 1* (*CXCR1*) and *c-x-c motif chemokine receptor 2* (*CXCR2*) were identified in the same region associated with FEC through ROH analysis [24] and hemoglobin (HGB) traits after an ssGWAS analysis using haplotype information [30]. Ahbara et al. [24] also identified *CXCR1* and *CXCR2* genes enriched (*p* < 0.001) in the term cluster “Cytokine–cytokine receptor interaction.” Both genes produce a cell surface receptor that binds with the chemokine CXCL-8 (IL-8), which controls leucocyte (i.e., neutrophil) recruitment and activation [66]. Based on the current literature, the role of CXCL-8 in the host inflammatory response to GINs has not been described. However, in humans, high levels of CXCL-8 were found in inflammatory bowel disease profiles when compared to healthy mucosa. Additionally, *CXCR1* receptors were found to be highly regulated, which indicates that CXCL-8 may be mediating the inflammatory response in the gastrointestinal (GI) tract of humans [67].

Berton et al. [29,30] and Farahani et al. [46] identified the *c-x-c motif chemokine receptor 4* (*CXCR4*) gene in regions found to be associated with RBC, FEC, and PLT and resistance to GINs, respectively. Farahani et al. [46] performed a GWAS using a Bayesian approach and haplotype information using 12 traits related to resistance to GINs in a population of Scottish Blackface lambs. Sheridan et al. [68], using breast cancer cell lines with low and high levels of *CXCR4* gene expression, found that six major histocompatibility complex (MHC) class II genes were downregulated in cell lines with high levels of *CXCR4* expression. Considering the likely importance of the MHC class II genes in the immune response to infection, the relationship between them and *CXCR4* should be studied further. In addition, *CXCR4* controls the migration of T-cells and, combined with the *c-c chemokine receptor type 5* (*CCR5*) receptor, plays a role in the chemokine-mediated co-stimulation of T-cells [69].

The *ubiquitin protein ligase e3 component n-recognin 3* (*UBR3*) gene encodes a protein called E3 ubiquitin-protein ligase that uses ubiquitination to control several biological processes [70]. Ubiquitination is known to play a role in several activities of the immune system, such as autophagy, phagocytosis of microbial pathogens, antigen presentation, T-cell activation, and B-cell signaling [71]. This gene has been identified in regions associated with *H. contortus* resistance [40] and the PLT trait [30].

The autophagy process is known to have other important functions within the immune system, such as contributing to cytokine regulation and antigen presentation in dendritic cells [72]. Terawaki et al. [73] found the *run and fyve domain containing 4* (*RUFY4*) gene positively influencing the autophagy process and helping prevent infection by *Brucella abortus*. The *RUFY4* gene was identified by Ahbara et al. [24] in regions associated with FEC and by Berton et al. [30] as associated with HGB.

The *dipeptidyl peptidase 4* (*DPP4*) gene was found in regions associated with RBC, PLT [29], and *H. contortus* resistance [40] traits. This gene appears to be expressed in T-cells, NK cells, and B-cells. In addition, it has been found to be involved in T-cell development and stimulation and plays a role in several immune responses and inflammatory diseases [74].

Regions associated with FEC in Tunisian sheep and with HGB in Santa Inês sheep harbored the *solute carrier family 11 member 1* (*SLC11A1*) gene. This gene encodes an ion transportation membrane protein but also plays a role in the defense against pathogens. Polymorphisms in this gene have been associated with a lower incidence of bovine tuberculosis, resistance to Brucellosis, and resistance to Salmonellosis in chickens [75]. According to Pires et al. [76], different patterns of methylation for island 2 on the *SLC11A1* gene may also be associated with horses presenting cyathostomin FEC, an endoparasite that lives in the large intestine of horses. The *SLC11A1* gene acts on macrophage function by increasing the synthesis of keratinocyte chemoattractant (KC) chemokine, MHC class II molecules, IL-1β, tumor necrosis factor (TNF) α, and inducible nitric oxide synthase (iNOS), which are fundamental in intramacrophage infection [77].

The *signal transducer and activator of transcription 4* (*STAT4*) gene was identified in regions associated with resistance to GINs [46] and HGB and RBC [30]. This gene is involved in several steps of the early immune response. It plays a role in IFN-γ production, differentiation of Th1 cells, and several cytokine signaling pathways [78,79]. In mice lacking the *STAT4* gene, macrophages displayed a decreased ability to secrete TNFα, IL-1β, IL-12, and nitrite [79]. It appears that an optimum immune response against helminth parasites relies on the presence of the STAT4 gene [79].

Eight QTLs overlapped among studies on OAR 2 (193058, 193020, 12898, 12883, 12938, 19801, 19789). QTL 12898 was found to have the highest overlapping between studies and traits. This region was initially associated with *Trichostrongylus* spp. adults and late-stage larvae counts found in the abomasum collected at slaughter at the end of the second parasite challenge by Crawford et al. [35]. The authors performed a QTL detection study through least squares interval mapping using microsatellites distributed all over the genome in resistant and susceptible lines of F1 Romney × Coopworth ewes. This QTL was later associated with FEC [24], PCV and FAMACHA© [25], FEC at 28 days after deworming [59], and resistance to GINs [46].

QLTs 193058 and 193020 were discovered by Riggio et al. [61] in a study using two different methodologies to identify regions associated with antigen-specific IgA levels and FECs of strongyles and *Nematodirus* spp. using a 50K SNP panel in naturally exposed Scottish Blackface Merino. The authors applied a GWAS fitting a mixed model and regional heritability mapping (RHM). QLTs 193058 and 193020 were associated with *Nematodirus* spp. average FEC and *Nematodirus* spp. FEC at 20 weeks of age, respectively [61]. Both QTLs were also found to be associated with average FEC in a study by Pickering et al. [19].

QTLs 12883 and 12938 were associated with average FEC in a population of different breeds and crossbred animals [19]. QTL 12883 was first discovered by Davies et al. [36] to be associated with *Nematodirus* spp. FEC at 20 weeks of age, where regression methodologies were applied using microsatellites distributed in specific chromosomes (1, 2, 3, 5, 14, 18, 20, and 21) in a study with Scottish Blackface sheep exposed to a mixed species of GINs while grazing. QTL 12938 was associated with the first artificial challenge on animals at 6 months of age [57]. Marshall et al. [57] performed two artificial challenges with *H. contortus* after deworming the animals at 6 and 13 months of age. Linkage-based and interval mapping using logistic regression (LR) for within-family analysis and interval mapping using maximum likelihood (ML) for across-family were performed between FEC phenotypes and microsatellites distributed through all chromosomes, including the X chromosome.

Marshall et al. [38] dewormed lambs (double backcross of Red Massai × Dorper) and maintained their grazing under natural exposure to GINs for 6 to 8 weeks. After that, the animals were dewormed again, and when the physiological parameters returned to normal levels, the animals were artificially challenged bi-weekly with *H. contortus* for 5 to 7 weeks. Association analyses were performed using four different models between the phenotypes and microsatellites distributed through all chromosomes (except OAR 24). From the results, these authors identified two novel QTLs (19789 and 19801) within the same region on OAR 2 using two different models that were associated with FEC. Both QTLs were also found to be associated with resistance to GIN traits in Scottish Blackface lambs [46].

### 2.3. Chromosome 3

Located within OAR 3 is *interferon γ* (*IFNG*), which is a major gene playing several roles in the immune system. Due to its importance in the immune system response, several association studies focused on studying OAR 3, hoping to identify SNPs within or surrounding this gene that may be associated with desirable phenotypes for resistance or resilience to GINs in sheep [33,34,36,62]. Coltman et al. [34] aimed to identify polymorphisms within *IFNG* that were associated with FEC and IgA levels in a free-living population of Soay sheep that were naturally infected and measured these phenotypes at 4 and 16 months of age. The authors found a reduction in FEC associated with an allele within the *IFNG* gene. However, no other study has associated resistance to GINs with *IFNG*. The lack of evidence associating *IFNG* with resistance to GINs does not exclude its possible role in the immune response, since this cytokine is a potent driver of cell-mediated immunity [80].

The *cyclin-dependent kinase inhibitor 1B* (*CDKN1B*) gene was identified in regions associated with FEC [24], HCT [29], and PLT [30]. *CDKN1B* is responsible for T-cell cycle regulation. This gene is also responsible for functions such as inducing apoptosis and impeding the multiplication of effector and memory CD4^+^ T-cells, modulating the homeostasis of CD8^+^ T cells, and controlling the proliferation of memory cells [81].

Kunimura et al. [82] identified the *endothelial pas domain 1* (*EPAS1*) gene as playing a major role in IL-31 production by CD4^+^ T-cells. Perrigoue et al. [83] identified IL-31 and its receptor IL-31R as controlling the Th2 cell response in the intestine of mice after parasite infection. This gene was identified by Álvarez et al. [25] to be associated with FAMACHA© and by Berton et al. [29] to be associated with HCT.

The *suppressor of cytokine signaling 2* (*SOCS2*) gene was identified in regions associated with average FEC [6] and under selection for *H. contortus* resistance when resistant and susceptible breeds were compared [44]. The SOCS2 protein regulates innate and adaptive immune responses. This protein regulates cellular response to cytokines and was identified as playing roles in the production and differentiation of Th1, Th2, T-helper 17 (Th17), and Treg cells [84]. It was also observed to be associated with the development or function of several other systems [85].

Twelve QTLs were identified among studies within OAR 3. QTL 12882 was identified by Davies et al. [36] to be associated with *Nematodirus* spp. FEC at week 16. Later, this QTL was identified by Pickering et al. [19] to be associated with estimated breeding values (EBVs) for dag score at 8 months and by Ahbara et al. [24] as associated with FEC.

Beh et al. [60] discovered QTL 12885 to be associated with *T. colubriformis* FEC after the second round of artificial challenges in Merino sheep. Farahani et al. [46] also identified this QTL as associated with resistance to GIN traits. QTL 12890, initially revealed by Davies et al. [36], was associated with IgA activity, and it was also associated with summer and fall FEC [19]. Davies et al. [36] also identified QTL 12891 as associated with strongyle FEC at 24 weeks of age, and later, this QTL was associated with IgA levels by Atlija et al. [27].

QTLs 12897, 12939, and 12940 were revealed by Marshall et al. [57] as associated with two measurements of FEC after the first artificial challenge with *H. contortus* in animals with 6 months of age (12897 and 12939) and with two measurements of FEC after the second artificial challenge with *H. contortus* in animals with 13 months of age (12940). QTL 12897 was later associated with FEC [24] and resistance to GINs [46]. QTL 12939 was identified by Estrada-Reyes et al. [59] as associated with an SNP presenting a significant additive effect for initial FEC measurement, and the same authors also identified QTL 12940 as associated with SNPs presenting a significant recessive effect for initial FEC measurement and FEC at 28 days.

An association study using a maximum likelihood approach with microsatellites spread all over the genome was performed in a backcross F1 (Merino × Romney) × Merino population [37]. This study performed two artificial challenges with *T. colubriformis*, followed by a third infection with *H. contortus*. The authors considered the average FEC measured at days 21, 28, and 35 after each infection and the eosinophil difference between days 0 and 28 after each *T. colubriformis* challenge and between days 0 and 35 after the *H. contortus* challenge. The authors identified two novel QTLs (14155 and 14156) associated with average FEC after the first infection and eosinophil differences after the second challenge, respectively. QTL 14155 was also identified by Ahbara et al. [24] as associated with FEC, and Pickering et al. [19] associated QTL 14156 with de-regressed breeding values (BVs) of FEC measured during the summer and autumn seasons.

An association analysis using microsatellites and a least squares regression framework was performed in a double backcross population (Red Massai × Dorper ewes × F1 Red Massai × Dorper sires) [39]. The authors revealed that QTL 16023 was associated with the average of two FEC measurements collected one day apart at the end of grazing exposure. This QTL was also identified by Ahbara et al. [24] as associated with FEC.

Matika et al. [62] investigated chromosomes 3 and 14 to determine if QTLs were segregating in Texel and Suffolk breeds. The authors performed half-sib regression interval mapping techniques to find QTLs associated with *Nematodirus* spp. and strongyles FECs in animals around 20 weeks of age. The analysis was performed using microsatellite markers on OAR3 and OAR14. The authors identified QTL 17188 on OAR 3, associated with *Nematodirus* spp. FEC. The QTL was later associated with FEC in Tunisian sheep by Ahbara et al. [24].

The last QTL (193054) on OAR 3 identified in this review was initially associated with IgA activity [61]. Riggio et al. [61] used two approaches to identify regions in the genome associated with IgA activity and FECs of *Nematodirus* spp. and strongyles in Scottish Blackface lambs. The authors used genome-wide association analyses, fitting mixed models with both fixed and polygenic effects to each trait, and RHM methodology using the 50K SNP chip in their analysis. Later, this QTL was associated with two traits: de-regressed BVs of FEC measured in the summer and autumn [19].

### 2.4. Chromosome 6

On OAR 6, six genes were identified as overlapping between studies related to immune response. The *complement factor I* (*CFI*) gene encodes the Factor I protein responsible for the cleaving of C3b and C4b, which inhibits the formation of the component C3, an important component in the regulation of the complement system [86]. The complement system is an important element of the innate adaptive immune system and can be activated by three different pathways, of which C3 is necessary for the activation of all three [87]. The C3 component was identified as necessary, in both innate and acquired immune systems, for the larvae-killing process in mice infected with *Strongyloides stercoralis* [87]. In addition, the *CFI* gene was identified as overexpressed in the resistant Morada Nova lamb abomasum mucosa, and this suggests that it may be playing a role in the early response against *H. contortus* infection [88]. This gene was identified in two different studies: the first associated with FEC at 28 days [41] and the second with *H. contortus* resistance [40].

The *cholinergic receptor nicotinic α-9 subunit* (*CHRNA9*) gene is responsible for the nicotinic acetylcholine receptor (nAChR) subunit α-9. This gene was associated with FEC [24], RBC, and white blood cell count (WBC) [30]. This gene seems to trigger an anti-inflammatory response, and it can be hijacked by pathogens in order to evade the immune response [89]. The nAChR subunit α-9 was identified as expressed in T and B-cells [90] and reduced or enhanced in Th cells and murine-induced Treg cells [91], which indicates that this receptor may be playing a role in the immune system and indicates further studies should be performed to better understand its role in immune response in sheep.

The *c-x-c motif chemokine ligand 9* (*CXCL9*), *c-x-c motif chemokine ligand 10* (*CXCL10*), and *c-x-c motif chemokine ligand 11* (*CXCL11*) genes encode chemokines that conduct several functions, including binding to the CXCR3 receptor and regulating leukocyte migration to the inflammation site, activation, and differentiation [92]; rejecting organs after transplantation; serving as biomarkers to identify and monitor patients after transplantation [93]; and liver tissue inflammation response after ischemia/reperfusion injury [94] and several other diseases. These chemokines are induced by IFN-γ and can be largely found in the intestine epithelium [95]. *CXCL9* and *CXCL11* were identified as expressed in the gut tissue of mice 19 days after infection by *Trichuris muris* [96]. *CXCL9* was also identified as upregulated in the intestine tissue of sheep after four hours of infection by *Echinococcus granulosus* eggs [97]. According to Cliffe et al. [98], the chemokine *CXCL10* may control the rate of cell turnover in mice infected with *Trichuris trichiuria*, and this may influence the expulsion of the parasite from the intestine. The *CXCL9* gene was associated with FEC [27], WBC, and FAMACHA© [30]. The *CXCL10* gene was associated with FEC [27], PLT [29], neutrophil count at day zero [41], WBC, and FAMACHA© [30]. The *CXCL11* gene was associated with FEC [27], WBC, and FAMACHA© [30].

Berton et al. [29] identified the *lymphoid enhancer binding factor 1* (*LEF1*) gene associated with HGB, and Niciura et al. [40] identified this gene as associated with *H. contortus* resistance. This gene plays a role in the thymocyte survival, endurance of CD8^+^ T memory cells, and differentiation of mature CD4+ T cells to Th2 and Th17 cells [99].

The *ras homolog family member h* (*RHOH*) gene produces a protein that is important in the development of T lymphocytes [100]. This gene is also involved in T-cell receptor (TCR) and pre-TCR signaling regulation during T-cell development [101]. TCRs are important in binding with foreign antigens, which leads to the activation of T-cells [102]. Gu et al. [103] demonstrated that mice deficient in *RHOH* presented diminished TCR, which led to T-cell deficiency. The study highlighted that *RHOH* is crucial for thymocyte development and TCR signaling. Ahbara et al. [24] associated this gene with FEC, and Berton et al. [30] associated this gene with WBC and RBC.

The *ubiquitin-like modifier-activating enzyme 6* (*UBA6*) gene seems to also be responsible for the initiation and transfer of the ubiquitin protein to the next steps [104]. The importance of the ubiquitination process was discussed earlier in this review. Lee et al. [105] identified the *UBA6* gene controlling the production of IFN-γ in CD4^+^ and CD8 T cells. This gene was associated with FEC [27] and PLT [29,30].

Three QTLs were identified among studies on OAR 6. QTL 16024 was first identified by Silva et al. [39] as associated with the average FEC of two measurements taken one day apart at the end of natural exposure. This QTL was also associated with FEC [24,27] and resistance to GINs [46]. QTL 12942 was revealed by Marshall et al. [57] as associated with two measurements of FEC taken on days 25 and 31 after the second artificial challenge with *H. contortus* and associated with average FEC by Pickering et al. [19]. QTL 13988 was discovered by Gutiérrez-Gil et al. [58] as associated with FEC at day 60 after drenching and natural exposure to GINs, and later associated with FEC after natural exposure by Atlija et al. [27].

### 2.5. Chromosome 7

Several genes on OAR 7 overlapped among different studies. However, only a few were identified as playing roles in the immune system. The *fhc and mu domain containing endocytic adaptor 1* (*FCHO1*) gene was pointed out as coordinating T-cell development and function [106]. The *janus kinase 3* (*JAK3*) gene plays essential roles in the immune system, such as in the function and maturation of B and T-cells [107], and mutations in this gene were associated with severe combined immune deficiency in humans [108]. The *microtubule associated protein 1s* (*MAP1S*) gene seems to interfere with Toll-like receptor (TLR) singling pathways [109] and may play an important role in neutrophil differentiation [110]. All three genes were associated with FEC by Atlija et al. [27] and with FAMACHA© by Berton et al. [30].

Genes such as *smad family member 3* (*SMAD3*) and *interleukin 25* (*IL25*) seem to play important roles in the mucosa immune response and should be considered for further studies. The *SMAD3* gene appears to play several roles in the immune system [111], including latent importance in the installation of the immune response in the mucosa [112]. The *IL25* gene codes for IL-25, which is a major constituent of the gastrointestinal tract’s protective immunity [113]. Both genes were associated with FEC [27,30]. The last gene identified was *egf like repeats and discoidin domains 3* (*EDIL3*). This gene function in the immune system was largely discussed by Becker et al. [23], and variants inside this gene were associated with different FEC by the same authors and with WBC by Berton et al. [30].

Three QTLs overlapped among association studies. QTLs 12944 and 12945 were first described by Marshall et al. [57] as associated with FEC after the first and second artificial challenges, respectively. Similarly, both QTLs were associated with FEC measured in the summer [19]. QTL 12964 was described by Marshall et al. [57] as associated with FEC after the second artificial challenge. Later, this QTL was associated with FEC [27] and FEC measured in the summer [19].

### 2.6. Chromosome 8

Among the several candidate genes identified as overlapping among association studies, two genes showed some relationship with the immune system. The *cd109 molecule* (*CD109*) gene was associated with FEC [27], RBC, HCT, and HGB [29], and *H. contortus* resistance [40]. *CD109* seems to be involved in the establishment of cutaneous inflammation, and on dendritic cells, the product of this gene may play a role in airway hyperreactivity and eosinophilic airway inflammation [114,115]. According to Taki et al. [116], this gene was observed to play a role in the regulation of transforming growth factor β (TGF-β) in lung cancer. Considering this gene’s role in mucous tissue, further studies should be considered to understand its role in the GIN immune response. The *sumo specific peptidase 6* (*SENP6*) gene regulates TLR, which plays a role in pathogen recognition and activation of the immune system [117,118]. This gene was associated with FEC [27], RBC, HCT, and HGB [29].

Five QTLs overlapping among studies were identified within chromosome eight. Silva et al. [39] found QTL 16025 was associated with average FEC, and later this QTL was associated with IgA levels [27] and FEC [24]. QTLs 193049 and 193051 were identified by Riggio et al. [61] as associated with strongyles FEC at 24 weeks of age, and later, both QTLs were associated with BVs of dag scores at eight months of age [19]. QTLs 12899 and 12900 were discovered by Crawford et al. [35] as associated with counts of adults and late-stage larvae of *Trichostrongylus* spp. at the end of the second parasite challenge found in the abomasum and small intestine, respectively. Later, both QTLs were associated with FAMACHA© measured at the beginning of the trial [59], FEC [24], FEC and PCV [25], IgA levels [27], and resistance to GINs [46].

### 2.7. Chromosome 9

On OAR 9, no candidate genes playing roles in the immune system were identified as overlapping among studies. Only QTL 16026 was associated with FEC and overlapped among different studies: Silva et al. [39] and Atlija et al. [27].

### 2.8. Chromosome 10

Among the several genes overlapping between studies within chromosome 10, four genes may be related to the immune response. The *growth arrest specific 6* (*GAS6*) gene was suggested as a wide controller of the innate immune system [119]. Estrada-Reyes et al. [47] investigated the copy number variant (CNV) associated with *H. contortus* FEC, PCV, FAMACHA©, BCS, and average daily gain in Florida Native sheep at two different sampling time points. First, on drench day, when animals around three to five months old were drenched and released for grazing and natural infection, and at a second timepoint at 38 days after drenching. The authors associated the *GAS6* gene with FEC and PCV measured on drench day and PCV measured 38 days later. This gene was also associated with RBC [29].

The *glucosaminyl (n-acetyl) transferase 3*, *mucin type* (*GCNT3*) encodes an important protein in the catalyzation of mucin glycoproteins. Mucin is a very important secretion produced during worm infection as a defense mechanism of the immune system to expel the worms [120]. During the *Ostertagia ostertagi* infection, *GCNT3* was found to be upregulated in the bovine abomasum tissue [120]. This gene was also associated with *H. contortus* resistance [40] and WBC [30].

*Poly (ADP-Ribose) Polymerase 2* (*PARP2*), in association with the *PARP1* gene, was identified as playing a role in the maintenance of T-cell homeostasis [121]. The *PARP2* gene was associated with FEC [27,30]. The remaining gene, *Telomerase Associated Protein 1* (*TEP1*), can build an immune response in mosquitoes against parasites, and it should be considered for further studies involving sheep immune responses against GINs. The protein produced by the *TEP1* gene attaches to the surface of the parasite and identifies it as needing to be killed, preventing the parasite from occupying the gut epithelium [122]. The *TEP1* gene was identified as associated with FEC in refs. [27,30].

Riggio et al. [61] discovered that QTL 193038 was associated with strongyle FEC at 24 weeks old. Estrada-Reyes et al. [47] also identified the same QTL associated with *H. contortus* FEC and PCV measured on drench day and 38 days later. The second QTL (13989) was initially identified by Gutiérrez-Gil et al. [58] as associated with FEC on day 60. Later, this QTL was associated with FEC [24], IgA levels [27], FEC at 28 days [59], and resistance to GINs [46].

### 2.9. Chromosome 11

On chromosome eleven, several relevant candidate genes have been identified to be associated with different traits measuring resistance to GINs. The *cd7 molecule* (*CD7*) gene encodes the CD7 molecule that could be participating in the T-cell activation process [123]. This gene overlapped among studies associated with IgA levels [27] and with FEC measured 38 days after drenching [47].

The *integrin subunit β 3* (*ITGB3*) gene is known to participate in several immune system responses, including wound healing [124]. Atlija et al. [27], Berton et al. [29], and Estrada-Reyes et al. [47] associated the *ITGB3* gene with IgA levels, WBC, and FEC measured 38 days after drenching, respectively.

The *nuclear receptor corepressor 1* (*NCOR1*) gene was identified as regulating CD4^+^ T cells, affecting the migration of some types of Th cells, thymocyte survival, and peripheral T-cell quantities [125]. Niciura et al. [40] and Berton et al. [30] associated this gene with *H. contortus* resistance and PLT, respectively.

The *Nitric Oxide Synthase 2* (*NOS2*) gene encodes an enzyme called nitric oxide synthase (NOS) responsible for nitric oxide (NO) production. Nitric oxide serves an important role in the defense against pathogens, including playing beneficial roles against helminthic infections [126]. The NO was identified as influencing the growth, reproductive organ formation, and egg development of *Schistosoma japonicum* [127]. In addition, the *NOS2* gene was found upregulated in the small intestine of bovines during the reinfection of *Cooperia oncophora* [128]. The gene *NOS2* was associated with *H. contortus* FEC at 38 days after deworming [47] and was identified under selection for *H. contortus* resistance when resistant and susceptible breeds were compared [44].

The genes *signal transducer and activator of transcription 3* (*STAT3*) and *signal transducer and activator of transcription 5b* (*STAT5B*) are part of the signal transducer and activator of transcription (STAT) family, which is critical to the immune system response [129]. The *STAT3* gene was highlighted as playing important roles in both innate and adaptive immune responses [130]. Wittkopf et al.’s [131] results showed that mice without this gene showed more susceptibility to intestinal bacterial infection. Estrada-Reyes et al. [41] and Estrada-Reyes et al. [47] associated the *STAT3* gene with *H. contortus* FEC measured at the beginning of the experiment and 38 days after deworming, respectively. In contrast, Estrada-Reyes et al. [47] associated *STAT5B* with *H. contortus* FEC at 38 days after deworming, and Estrada-Reyes et al. [44] identified it under selection for *H. contortus* resistance when resistant and susceptible breeds were compared. The absence of this gene may severely affect immunological response and Treg cell viability [129,132].

Estrada et al. [44] revealed several novel QTLs (180504, 180505, 180516, 180528, 180529, 180530, 180541, 180542, 180543, 180544, 180545, 180551, 180556, and 180557) under selection for *H. contortus* resistance within the OAR 11 when resistant and susceptible sheep breeds were compared. Later, all QTLs were associated with *H. contortus* FEC at 38 days after deworming [47]. Marshall et al. [57] discovered that QTL 12949 was associated with *H. contortus* FEC after the first artificial challenge in animals six months of age. The same QTL was later associated with the EBV of the FEC measured during the summer and the EBV of an adult FEC generated based on estimations of phenotypic and genotypic correlation, among other traits [19]. The last QTL (12901) identified as overlapping among studies was initially associated with counts of *Trichostrongylus* spp. adults and late-stage larvae found in the small intestine of slaughtered animals at the end of the second parasite challenge by Crawford et al. [35]. This QTL was associated with FEC, *H. contortus* FEC 38 days after deworming, and resistance to GINs [24,46,59].

### 2.10. Chromosome 12

Three genes overlapping among studies presented some relationship with functions or regulation of the immune system on OAR 12. The *ephrin b2* (*EFNB2*) gene was previously found to play a role in the development and function of T-cells [133]. Estrada-Reyes et al. [47] associated the *EFNB2* gene with *H. contortus* FEC and PCV measured at the beginning of the experiment and 38 days after deworming, and Berton et al. [30] associated the same gene with HCT, HGB, and RBC.

The *toll-like receptor 5* (*TLR5*) gene encodes a receptor that recognizes flagellin and plays a role in activating the innate and adaptive immune response, playing an important role in intestinal immune homeostasis [134]. *TLR5* plays a role in the recognition of flagellin present in the flagella of bacteria and may have the capacity to increase the immune response to trichomoniasis infections [135]. This gene was associated with PLT [29] and FEC [31], and we believe further investigation regarding its function in the parasite immune response should be performed.

The last relevant gene is *tnf superfamily member 13b* (*TNFSF13B*). This gene was associated with *H. contortus* FEC at the beginning of the experiment and at 38 days after deworming [47] and with HCT, HGB, and RBC [30]. This gene encodes the elevated B-cell-activating factor (BAFF), which is crucial in B-cell development and homeostasis [136] and memory B-cell survival [137]. A polymorphism in this gene was positively associated with IgG levels against *Ascaris lumbricoide*, a nematode found in the human intestine [138].

Four QLTs were found to overlap among studies. QTL 193042 was initially associated with *Nematodirus* spp. FEC at 20 weeks of age [61] and later with the EBV of the FEC measured in the autumn [19]. QTL 126086 was also associated with the EBV of the FEC measured in the autumn by Pickering et al. [19]. However, this QTL was discovered to be associated with the average of two FEC measurements after the first round of artificial challenge with *H. contortus* at 3 months of age by Sallé et al. [63], where the association analysis was performed using the least squares regression framework in a backcross population between Martinik Black Belly and Romane breeds (resistant vs. susceptible breeds). QTL 95627 was revealed by Atlija et al. [27] as associated with IgA levels and later was associated with FEC [24] and resistance to GINs [46]. The last QTL (12889) found overlapping among studies was uncovered by Beh et al. [60] as associated with the FEC of *T. colubriformis* after the second round of artificial challenge and with IgA levels by Atlija et al. [27].

### 2.11. Chromosome 13

Among the few candidate genes found to overlap between studies on OAR 13, the gene *interleukin 2 receptor subunit α* (*IL2RA*) encodes one subunit of the IL-2 receptor that is involved in controlling Treg cells [139]. This gene expression was above control levels in resistant sheep following the *T. colubriformis* artificial challenge and in resistant sheep following the *H. contortus* challenge [140]. The *IL2RA* gene was associated with WBC, PLT [29], and RBC [30], and was identified under selection for *H. contortus* resistance when resistant and susceptible breeds were compared [44].

QTL 16027 was identified as overlapping among studies performed by Silva et al. [39] and Álvarez et al. [25]. Silva et al. [39] revealed this QTL to be associated with the average of two FEC measurements taken one day apart at the end of the grazing challenge, and Álvarez et al. [25] found this QTL to be associated with FAMACHA©.

### 2.12. Chromosome 14

Candidate genes overlapping among studies associated with immune response or resistance to GINs were not identified on OAR 14. However, three QTLs were identified. Davies et al. [36] uncovered QTLs 12892, 12893, and 12894 as initially associated with *Nematodirus* spp. FEC at 16 weeks of age, *Nematodirus* spp. average FEC, and *Nematodirus* spp. FEC at 20 weeks of age, respectively. Later, QTL 12892 was associated with FEC [24] and RBC [29], QTL 12893 was associated with FEC [24], and QTL 12894 was associated with resistance to GINs [46].

### 2.13. Chromosome 15

Two genes and one QTL were identified on OAR 15 as associated with traits measuring resistance to GINs. The *cullin 5* (*CUL5*) gene was pointed out as playing a role in CD4^+^ T-cell differentiation. The lack of this gene in T-cells is known to lead to mild Th2 cell-mediated inflammation that is aggravated with age [141]. This gene was associated with *H. contortus* resistance [40] and FAMACHA© [30].

The second gene, *mucin 15 cell surface associated* (*MUC15*), is part of a subfamily that produces cell surface mucin. The stomach and small and large intestines produce a layer of mucus that is important to protect the mucosa against commensal microorganisms, viruses, bacteria, and eukaryotic pathogens [142]. Mucin is likely to be the first barrier against pathogens and prevents the pathogen from attaching to the inner layers [142]. Both innate and adaptative immune systems can regulate the production of mucin through different mechanisms [142]. In this review, this is the second gene reported to play a role in the production of mucin, which emphasizes the importance of this mechanism in the immune response. This gene was identified as associated with traits measuring resistance to GINs [6], initial FEC [41], and WBC at 28 days [41].

Only QTL 16029 was identified among studies on OAR 15 as associated with traits measuring resistance to GINs in sheep. This QTL was revealed initially by Silva et al. [39] to be associated with FEC average and later by Atlija et al. [27] and Pickering et al. [19] as associated with IgA levels and dag scores at three and eight months of age, respectively.

### 2.14. Chromosome 17

OAR 17 was home to several genes that play a role in the immune response to GINs. The *dynein light chain lc8-type 1* (*DYNLL1*) gene was identified as overlapping among studies on OAR 17. This gene may play multiple roles in inflammation and immune response. It has been previously found to participate in the development of different lineages of B-cells [143] and may act as a regulator of NF-κB and *TLR4* signaling [144]. Atlija et al. [27] identified the *DYNLL1* gene as associated with IgA levels, and Niciura et al. [40] associated this gene with *H. contortus* resistance.

The *fibroblast growth factor 2* (*FGF2*) gene seems to coordinate with IL-17 to stimulate the genes to work to repair the damaged epithelium in colitis diseases [145], which could indicate the importance of *FGF2* in repairing the intestinal epithelium after parasite damage. This gene was associated with FEC [24,29] and FEC, PLT, HCT, HGB, and RBC [30]. Considering the number of studies and traits this gene overlapped with, it should be considered for further studies.

The *interleukin 15* (*IL15*) gene encodes IL-15. The role of this interleukin on the parasite immune response is not very clear; IL-15 was shown to be important in APCs and in the production of interleukin-12, interferon-γ, and NO [146]. Reinecker et al. [147] suggested that epithelial intestinal cells expressed IL-15 and may be using IL-15 in the differentiation and development of lymphocytes. Additionally, IL-15 seems to be an important element in the proliferation and homeostatic survival of memory T-cells [148]. The *IL15* gene was associated with IgA levels [27], FEC and WBC [29], and FEC [30].

The *phospholipase a2 group ib* (*PLA2G1B*) gene is important in the immune system response against GINs and should be considered in further studies. According to Entwistle et al. [149], the pancreatic phospholipase A2 encoded by this gene prevents larvae from developing into adult parasites, which is essential for establishing resistance against intestinal helminth in mice. Palma et al. [150] also stated that this protein interferes with the development of the parasite larvae into adults, contributing to increased resistance to parasites. PLA2G1B protein was found to be upregulated in resistant mice, and mice with the absence of this gene were unable to expel the parasites. According to the authors, the adaptive immune system is responsible for this gene expression. This gene was associated with IgA levels [27] and *H. contortus* resistance [40].

Mice with a knockout on the *set domain containing 7 histone lysine methyltransferase* (*SETD7*) gene presented resistance against *T. muris*, a nematode that affects mice. Apparently, the lack of the *SETD7* gene affects intestinal epithelial cell turnover, which makes this mechanism more effective in combating intestinal parasites when compared with the adaptative immune system [151]. Atlija et al. [27] identified this gene as associated with IgA levels, and Berton et al. [30] associated it with FEC.

Two QTLs (16031 and 95633) were identified as overlapping among studies. QTL 16031 was discovered by Silva et al. [39] as associated with average FEC. Later, it was associated with FEC and IgA levels [27] and resistance to GINs [46]. QLT 95633 was revealed by Atlija et al. [27] to be associated with IgA levels. Later, it was associated with FEC by Ahbara et al. [24] and resistance to GINs by Farahani et al. [46].

### 2.15. Chromosome 18

Among the few genes overlapping on chromosome 18, the *Interleukin 16* (*IL16*) gene was the only gene related to the immune system. IL-16, produced by the *IL16* gene, plays roles in the immune system such as T-cells, eosinophils, monocytes, and dendritic cell migration, among other functions [152]. This gene was associated with PLT [29] and RBC and HGB at day 28 of the experiment [41].

Among the QTLs identified as overlapping through studies, QTL 16037 was initially revealed by Silva et al. [39] as associated with PCV at the start of the challenge period. Álvarez et al. [25] also associated this QTL with the FAMACHA© score. QTL 19806 was discovered by Marshall et al. [38] as associated with the number of adult worms in the abomasum at necropsy time. Later, Ahbara et al. [24] associated this QTL with FEC. The last QTL (12965) was initially associated with an average of two FEC after the first artificial challenge with *H. contortus* at 6 months of age [57]. Later, Farahani et al. [46] associated this QTL with resistance to GINs.

### 2.16. Chromosome 20

On OAR 20, the *tumor necrosis factor* (*TNF*) gene, also known as TNF-α, may be associated with the immune response against GIN infection and seems to play a role in the expulsion of *T. muris* in mice through IL-13 regulation [153]. This gene was overall higher in expression in afferent and efferent intestinal lymph cells of resistant line sheep than in susceptible line sheep, which, according to the authors, may be the explanation for an increased acute inflammatory response in resistant lines [154]. This gene was identified in a study where the authors used LDLA to identify QTLs associated with FEC using a 50K SNP panel [32]. This association analysis study was performed on the offspring of a Sarda Dairy sheep population deriving from purebred Sarda and F1 Sarda × Lacaune animals. Casu et al. [32] associated this gene with FEC, and Estrada-Reyes et al. [41] associated the same gene with neutrophil count at day 0 of the experiment (10 days after drenched). No QTLs for GIN-related traits were identified as overlapping among studies for OAR 20.

### 2.17. Chromosome 21

On OAR 21, no gene of immune importance was identified as overlapping among studies. However, five QTLs were identified. QTL 14157 was uncovered by Dominik et al. [37] to be associated with a change in eosinophil numbers as the difference between day 0 and day 28 of the first *T. colubriformis* artificial infection. This QTL was also associated with FEC [24] and FEC and IgA levels [27]. Atlija et al. [27] revealed that QTL 95638 was associated with IgA levels [27], and later, Estrada-Reyes et al. [59] associated this QTL with *H. contortus* FEC at day 28 (38 days post-drenching). QTLs 126104, 126105, and 126106 were discovered by Sallé et al. [63] and associated with serum pepsinogen levels at day 0 (126104) and day 15 (126105 and 126106) after the first artificial challenge with *H. contortus* larvae. All three QTLs were also associated with FEC by Ahbara et al. [24].

### 2.18. Chromosome 22

On OAR 22, one gene and one QTL were identified as associated with traits measuring resistance to GINs. The *toll-like receptor 9* (*TLR9*) was associated with FEC by Carracelas et al. [31] and with WBC and PLT by Berton et al. [30]. As discussed, TLRs are important elements in pathogen recognition and activation of the immune system [118]. In addition to intracellular *TLR9* being responsible for the recognition of bacterial and viral molecular patterns, *TLR9* was also associated with the recognition of malaria, an intracellular parasite [118]. Kosik-Bogacka et al. [155] associated the increase in *TLR9* expression levels in the small and large intestines of rats with *Hymenolepis diminuta* infection. In addition, Ingham et al. [140] identified the *TLR9* gene as upregulated in resistant sheep after *H. contortus* and *T. colubriformis* challenges. Considering the literature description, this gene seems to be an interesting candidate for the response against parasite infection. The only QTL (95640) overlapping among studies was initially revealed by Atlija et al. [27] as associated with IgA levels, and later by Álvarez et al. [25] as associated with FAMACHA©.

### 2.19. Chromosome 23

On chromosome 23, no genes of importance to the immune system were identified as overlapping among studies. However, four QTLs were identified. QTLs 19808 and 19791 were revealed by Marshall et al. [38] as associated with FEC. QTL 19808 was associated with resistance to GINs [46] and FEC, HCT, HGB, and RBC [30], and QTL 19791 was associated with resistance to GINs [46]. QLTs 12902 and 12903 were discovered by Crawford et al. [35] as associated with total IgE in serum collected at the end of the second parasite challenge and IgG specific to *T. colubriformis* in serum collected 4 weeks after the start of the second field challenge, respectively. QTL 12902 was associated with IgA levels [27], and QTL 12903 was associated with IgA levels [27] and resistance to GIN [46].

### 2.20. Chromosome 24

The *rio kinase 3* (*RIOK3*) gene on chromosome 24 overlapped between the studies by Atlija et al. [27] and Berton et al. [30] associated with IgA levels and FEC, respectively. This gene has been highlighted as playing several roles in the immune system. Differentially expressed mRNA isoforms of this gene were identified as regulating inflammatory pathways in response to viral infection [156]. Also, this gene seems to regulate the type I interferon pathway [157,158]. No QTLs associated with traits measuring resistance to GINs in sheep were identified as overlapping between studies.

### 2.21. Chromosome 25

On chromosome 25, three QTLs (19810, 19811, and 126112) were identified as overlapping between studies. Marshall et al. [38] uncovered QTLs 19810 and 19811 associated with the total (adult and immature worms) count of worms at necropsy and the total of adult worms at necropsy, respectively. Both QTLs were later associated with the EBVs of two samples of FEC taken in the summer [19]. QTL 126112 was revealed by Sallé et al. [63] to be associated with PCV after the second challenge with *H. contortus*, with values corrected for PCV at day zero of the experiment. Later, Pickering et al. [19] associated this gene with the EBVs of two samples of FEC taken in the summer. In contrast, genes playing important roles in the immune response were not identified as overlapping between studies.

### 2.22. Chromosome 26

On the remaining chromosome, the *toll-like receptor 3* (*TLR3*) gene and six QTLs were identified as overlapping among studies. The *TLR3* gene encodes another intracellular TLR, TLR3 [159]. These receptors are present in dendritic cells and are responsible for microorganisms and parasite recognition, and possibly the Th1 response to infection, likely followed by the innate and adaptive immune responses [159]. This gene was associated with WBC [29] and FEC on day 28 of the experiment [41].

Marshall et al. [57] uncovered QTLs 19813, 19814, 19815, and 19816 associated with PCV, FEC at the end of the challenge, the total (adult and immature worms) count of worms at necropsy, and the total of adult worms at necropsy, respectively. All four QLTs were later associated with the EBVs of two samples of FEC taken in the summer [19]. QTL 19817 was also revealed by Marshall et al. [57] to be associated with the count of immature worms at necropsy. Pickering et al. [19] associated this QTL with three traits: the EBVs of two samples of FEC collected in the summer, the EBVs of two samples of FEC collected in the autumn, and the EBVs for average FEC estimated from genetic and phenotypic correlations with FEC measured in the summer and autumn. The last QLT (12962) was discovered by Marshall et al. [57] as associated with two FEC measurements after the second artificial challenge with *H. contortus* with animals at 13 months of age and was also associated with the EBVs of two samples of FEC taken in the summer [19].

### 2.23. Chromosomes 4, 5, 16, 19

On OAR 4, 5, 16, and 19, few genes were found to overlap between two or more studies. However, none of the genes played a direct role related to the immune system or in the regulation, expression, or signaling of any component in the immune system, based on the current literature. Also, QTLs overlapping among two or more studies were not identified as associated with traits measuring resistance to GINs in sheep.

Several genes that were not discussed here overlapped between the studies by Berton et al. [29,30] and Niciura et al. [40] within different chromosomes and traits. Some of these genes appeared to be involved with the immune system at some level, due to their association with different types of cancer or non-infectious diseases. For example, the gene *Mitogen-Activated Protein Kinase Kinase 3* (*MAP2K3*) is known to increase expression in skin diseases such as psoriasis, acne vulgaris, and atopic dermatitis, and the regulation of *MAP2K3* seems to be important for homeostasis [160]. Considering the number of regions and genes overlapping between the latter three papers and the studied breeds (Morada Nova and Santa Inês), which are naturally resistant to GIN infection, it may suggest a few of these genes play key roles in the defense against GIN infection and are important genes to be further investigated.

## 3. Conclusions

Despite being studied for several years, GIN resistance remains a poorly understood trait. Due to resistance being a polygenic and complex trait, multiple environmental, genetic, and experimental factors contribute to its variability, making it difficult to identify, validate, and apply key regulatory genes for this trait, which are consistent across populations and breeds.

Nonetheless, this review has summarized the findings of several studies that have identified genes and QTLs that overlap in their association with GIN resistance. While no single gene or QTL has been consistently identified across all studies, several important immune system genes have been identified. These genes are involved in various processes, such as T-cell activation, proliferation, differentiation, function, and adhesion; thymic T-cell proliferation, maturation, and survival; memory T-cell development and survival; as well as TLR signaling pathways and regulation.

In addition to these genes, genes that code for proteins important for mucin production, genes directly involved in mucin production, and genes that play a role in intestinal epithelium turnover have been identified. Furthermore, genes related to wound healing and repair have also been found to be associated with GIN resistance.

Finally, QTLs associated with traits measuring resistance were identified and could serve as potential candidate genes for further validation through additional studies.

## Figures and Tables

**Table 1:** Genes associated with traits measuring resistance to gastrointestinal nematodes in sheep breeds identified as overlapping between association studies.

Chromosome	Associated Gene	Associated Phenotype ^1^	Breed ^2^	Reference
1	*CCDC50*	Average FEC	Red Masaai × Dorper	[6]
		FAMACHA©	Santa Inês	[30]
		HCT	Santa Inês	[30]
		RBC	Santa Inês	[30]
	*CD86*	*H. contortus* resistance ^3^	Dorper, Katahdin, St. Croix	[44]
		RBC	Santa Inês	[29]
		PLT	Santa Inês	[29]
		HCT	Santa Inês	[29]
	*IL12RB2*	PLT	Santa Inês	[29]
		HCT	Santa Inês	[29]
		FEC	Florida Native	[41]
		FAMACHA©	Florida Native	[41]
		Total IgM level	Dorper, Katahdin, St. Croix	[45]
	*LEPR*	FEC	Corriedale	[31]
		RBC	Santa Inês	[29]
		PLT	Santa Inês	[29]
		HCT	Santa Inês	[29]
2	*ITGA6*	*H. contortus* resistance ^4^	Morada Nova	[40]
		PLT	Santa Inês	[30]
	*CXCR1*	FEC	Tunisan	[24]
		HGB	Tunisan	[24]
	*CXCR2*	FEC	Tunisan	[24]
		HGB	Tunisan	[24]
	*CXCR4*	RBC	Santa Inês	[29]
		FEC	Santa Inês	[30]
		PLT	Santa Inês	[30]
		Resistance to GINs ^5^	Scottish Blackface	[46]
	*UBR3*	*H. contortus* resistance ^4^	Morada Nova	[40]
		PLT	Santa Inês	[30]
	*RUFY4*	FEC	Tunisan	[24]
		HGB	Santa Inês	[30]
	*DPP4*	RBC	Santa Inês	[29]
		PLT	Santa Inês	[29]
		*H. contortus* resistance ^4^	Morada Nova	[40]
	*SLC11A1*	FEC	Tunisian	[24]
		HGB	Santa Inês	[30]
	*STAT4*	Resistance to GINs ^5^	Scottish Blackface	[46]
		HGB	Santa Inês	[30]
		RBC	Santa Inês	[30]
3	*IFNG*	FEC	Soay	[34]
	*CDKN1B*	FEC	Tunisian	[24]
		HCT	Santa Inês	[29]
		PLT	Santa Inês	[30]
	*EPAS1*	FAMACHA©	Djallonqe	[25]
		HCT	Santa Inês	[29]
	*SOCS2*	Average FEC	Red Masaai × Dorper	[6]
		*H. contortus* resistance ^3^	Dorper, Katahdin, St. Croix	[44]
6	*CFI*	FEC	Florida Native	[41]
		*H. contortus* resistance ^4^	Morada Nova	[40]
	*CHRNA9*	FEC	Tunisan	[24]
		RBC	Santa Inês	[30]
		WBC	Santa Inês	[30]
	*CXCL9*	FEC	Spanish Churra	[27]
		WBC	Santa Inês	[30]
		FAMACHA©	Santa Inês	[30]
	*CXCL10*	FEC	Spanish Churra	[27]
		PLT	Santa Inês	[29]
		Neutrophil count	Florida Native	[41]
		WBC	Santa Inês	[30]
		FAMACHA©	Santa Inês	[30]
	*CXCL11*	FEC	Spanish Churra	[27]
		WBC	Santa Inês	[30]
		FAMACHA©	Santa Inês	[30]
	*LEF1*	HGB	Santa Inês	[29]
		*H. contortus* resistance ^4^	Morada Nova	[40]
	*RHOH*	FEC	Tunisan	[24]
		WBC	Santa Inês	[30]
		RBC	Santa Inês	[30]
	*UBA6*	FEC	Spanish Churra	[27]
		PLT	Santa Inês	[29]
		PLT	Santa Inês	[30]
7	*FCHO1*	FEC	Spanish Churra	[27]
		FAMACHA©	Santa Inês	[30]
	*JAK3*	FEC	Spanish Churra	[27]
		FAMACHA©	Santa Inês	[30]
	*MAP1S*	FEC	Spanish Churra	[27]
		FAMACHA©	Santa Inês	[30]
	*SMAD3*	FEC	Spanish Churra	[27]
		FEC	Santa Inês	[30]
	*IL25*	FEC	Spanish Churra	[27]
		FEC	Santa Inês	[30]
	*EDIL3*	FEC	Katahdin	[23]
		WBC	Santa Inês	[30]
8	*CD109*	FEC	Spanish Churra	[27]
		RBC	Santa Inês	[29]
		HGB	Santa Inês	[29]
		HCT	Santa Inês	[29]
		*H. contortus* resistance ^4^	Morada Nova	[40]
	*SENP6*	FEC	Spanish Churra	[27]
		RBC	Santa Inês	[29]
		HCT	Santa Inês	[29]
		HGB	Santa Inês	[29]
10	*GAS6*	FEC	Florida Native	[47]
		PCV	Florida Native	[47]
		PCV	Florida Native	[47]
		RBC	Santa Inês	[29]
	*GCNT3*	*H. contortus* resistance ^4^	Morada Nova	[40]
		WBC	Santa Inês	[30]
	*PARP2*	FEC	Spanish Churra	[27]
		FEC	Santa Inês	[30]
	*TEP1*	FEC	Spanish Churra	[27]
		FEC	Santa Inês	[30]
11	*CD7*	Antigen-specific IgA activity	Spanish Churra	[27]
		FEC	Florida Native	[47]
	*ITGB3*	Antigen-specific IgA activity	Spanish Churra	[27]
		WBC	Santa Inês	[29]
		FEC	Florida Native	[47]
	*NCOR1*	*H. contortus* resistance ^4^	Morada Nova	[40]
		PLT	Santa Inês	[30]
	*NOS2*	*H. contortus* FEC	Florida Native	[47]
		*H. contortus* resistance ^3^	Dorper, Katahdin, St. Croix	[44]
	*STAT3*	*H. contortus* FEC	Florida Native	[41]
		*H. contortus* FEC	Florida Native	[47]
	*STAT5B*	*H. contortus* FEC	Florida Native	[47]
		*H. contortus* resistance ^3^	Dorper, Katahdin, St. Croix	[44]
12	*EFNB2*	*H. contortus* FEC	Florida Native	[47]
		PCV	Florida Native	[47]
		PCV	Florida Native	[47]
		HCT	Santa Inês	[30]
		HGB	Santa Inês	[30]
		RBC	Santa Inês	[30]
	*TLR5*	PLT	Santa Inês	[29]
		FEC	Corriedale	[31]
	*TNFSF13B*	*H. contortus* FEC	Florida Native	[47]
		PCV	Florida Native	[47]
		PCV	Florida Native	[47]
		HCT	Santa Inês	[30]
		HGB	Santa Inês	[30]
		RBC	Santa Inês	[30]
13	*IL2RA*	WBC	Santa Inês	[29]
		PLT	Santa Inês	[29]
		RBC	Santa Inês	[30]
		*H. contortus* resistance ^3^	Dorper, Katahdin, St. Croix	[44]
15	*CUL5*	*H. contortus* resistance ^4^	Morada Nova	[40]
		FAMACHA©	Santa Inês	[30]
	*MUC15*	Traits measuring resistance to GINs ^6^	Red Masaai × Dorper	[6]
		FEC	Florida Native	[41]
		WBC	Florida Native	[41]
17	*DYNLL1*	Antigen-specific IgA activity	Spanish Churra	[27]
		*H. contortus* resistance ^4^	Morada Nova	[40]
	*FGF2*	FEC	Tunisan	[24]
		FEC	Santa Inês	[29]
		FEC	Santa Inês	[30]
		PLT	Santa Inês	[30]
		HCT	Santa Inês	[30]
		HGB	Santa Inês	[30]
		RBC	Santa Inês	[30]
	*IL15*	Antigen-specific IgA activity	Spanish Churra	[27]
		FEC	Santa Inês	[29]
		WBC	Santa Inês	[29]
		FEC	Santa Inês	[30]
	*PLA2G1B*	Antigen-specific IgA activity	Spanish Churra	[27]
		*H. contortus* resistance ^4^	Morada Nova	[40]
	*SETD7*	Antigen-specific IgA activity	Spanish Churra	[27]
		FEC	Santa Inês	[30]
18	*IL16*	PLT	Santa Inês	[29]
		RBC	Florida Native	[41]
		HGB	Florida Native	[41]
20	*TNF*	FEC	Sarda Dairy × Lacaune	[32]
		Neutrophil count	Florida Native	[41]
22	*TLR9*	FEC	Corriedale	[31]
		WBC	Santa Inês	[30]
		PLT	Santa Inês	[30]
24	*RIOK3*	Antigen-specific IgA activity	Spanish Churra	[27]
		FEC	Santa Inês	[30]
26	*TLR3*	WBC	Santa Inês	[29]
		FEC	Florida Native	[41]

^1^ The phenotypes with the same acronyms may differ between the experimental design, type of infection, nematode species, and other aspects; refer to the study or main text for more details. ^2^ “×” indicates a crossbreed study, and “,” indicates several breeds used in the study. ^3^ *H. contortus* resistance, as defined by reference [40], is a phenotype measuring fecal egg count after artificial challenge with *H. contortus*, used to identify regions under selection for resistance to gastrointestinal nematodes. ^4^ *H. contortus* resistance, as defined by reference [38], is a phenotype obtained after ranking animals as extremely resistant or susceptible based on combined measurements of FEC, PCV, and live weight. ^5^ Resistance to GINs refers to the average of four replicates of fecal egg count that were collected at approximately 16, 20, and 24 weeks of age; however, the specific age at the time of sampling associated with the region was not specified by the authors. ^6^ Traits measuring resistance to GINs refers to one of multiple phenotypes (average fecal egg count under natural exposure, average packed cell volume, and average live weight) used by the authors; however, the specific trait associated with the region was not specified. FEC—fecal egg count, FAMACHA©—FAffa MAlan CHArt©, HCT—hematocrit, RBC—red blood cell, PLT—platelet, IgM—immunoglobulin M, HGB—hemoglobin, GINs—gastrointestinal nematodes, WBC—white blood cell count, IgA—immunoglobulin A, PCV—packed cell volume.

## Data Availability

The data presented in this study are available within the paper.
